# Don’t ask for fair treatment? A gender analysis of ethnic discrimination, response to discrimination, and self-rated health among marriage migrants in South Korea

**DOI:** 10.1186/s12939-016-0396-7

**Published:** 2016-07-19

**Authors:** Yugyun Kim, Inseo Son, Dainn Wie, Carles Muntaner, Hyunwoo Kim, Seung-Sup Kim

**Affiliations:** Department of Public Health Sciences, Graduate School of Korea University, Anam-ro, Seongbuk-gu Seoul, South Korea; Asiatic Research Institute, Korea University, Anam-ro, Seongbuk-gu Seoul, South Korea; National Graduate Institute for Policy Studies, 7-22-1, Roppongi, Minato-ku Tokyo, Japan; Bloomberg Faculty of Nursing and Dalla Lana School of Public Health, University of Toronto, Toronto, Canada; Department of Social & Behavioral Sciences, Harvard T.H. Chan School of Public Health, Boston, MA USA; Department of Environmental and Occupational Health, Milken Institute School of Public Health at George Washington University, Washington, DC, USA

**Keywords:** Ethnic discrimination, Response to discrimination, Gender difference, Marriage migrant, South Korea

## Abstract

**Background:**

Ethnic discrimination is increasingly common nowadays in South Korea with the influx of migrants. Despite the growing body of evidences suggests that ethnic discrimination negatively impacts health, only few researches have been conducted on the association between ethnic discrimination and health outcomes among marriage migrants in Korea. This study sought to examine how ethnic discrimination and response to the discrimination are related to self-rated health and whether the association differs by victim’s gender.

**Methods:**

We conducted two-step analysis using cross-sectional dataset from the ‘National Survey of Multicultural Families 2012’. First, we examined the association between perceived ethnic discrimination and self-rated health among 14,406 marriage migrants in Korea. Second, among the marriage migrants who experienced ethnic discrimination (*n*=5,880), we examined how response to discrimination (i.e., whether or not asking for fair treatment) is related to poor self-rated health. All analyses were conducted after being stratified by the migrant’s gender.

**Results:**

This research found the significant association between ethnic discrimination and poor self-rated health among female marriage migrants (OR: 1.53, 95 % CI: 1.32, 1.76), but not among male marriage migrants (OR: 1.16, 95 % CI: 0.81, 1.66). In the restricted analysis with marriage migrants who experienced ethnic discrimination, compared to the group who did not ask for fair treatment, female marriage migrants who asked for fair treatment were more likely to report poor self-rated health (OR: 1.21, 95 % CI: 0.98, 1.50); however, male marriage migrants who asked for fair treatment were less likely to report poor self-rated health (OR: 0.65, 95 % CI: 0.36, 1.04) although both were not statistically significant.

**Conclusions:**

This is the first study to investigate gender difference in the association between response to ethnic discrimination and self-rated health in South Korea. We discussed that gender may play an important role in the association between response to discrimination and self-rated health among marriage migrants in Korea. In order to prevent discrimination which could endanger the health of ethnic minorities including marriage migrants, relevant policies are needed.

**Electronic supplementary material:**

The online version of this article (doi:10.1186/s12939-016-0396-7) contains supplementary material, which is available to authorized users.

## Background

In industrialized Asian countries, especially Japan, Taiwan, and South Korea, migration has been increased since 1990s. Labor and marriage migration take the biggest proportion of migration to Japan, Taiwan, and South Korea because working age population has decreased due to low-birth rate [1]. The increase of marriage migrants could ingenerate the social issues including ethnic tensions, and it also happens in South Korea (hereafter Korea).

Since the early 1990s, Korea has seen an increase in the number of individuals migrating for marriage. Marriage migrants in Korea are about 250,000 in 2014 which hold about 15 % of total migrants in Korea. China (41.4 %), Vietnam (26.4 %), Japan (8.1 %), and the Philippines (6.9 %) are major regions of origin for marriage migrants in Korea [2]. At the same time, marriage migrants from North America or Western Europe also reside in Korea although they take a small proportion [3].

**Fig. 1:**
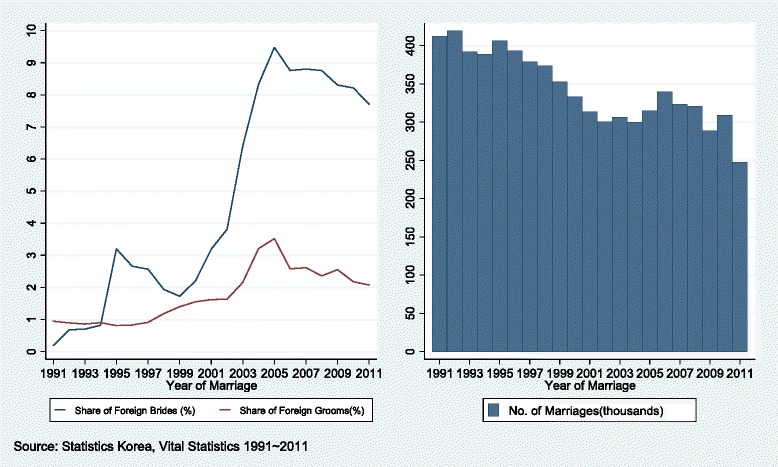
Trends of international marriage in South Korea, 1991–2011

In Korea, 85 % of marriage migrants are female, and they take around 8 % of total brides from the middle of 2000s (Fig. 1). The key factor affecting the supply side of female marriage migrants is global economic disparity [4]. Women in low income countries with poor economic background might seek better socioeconomic position through marriage migration, and marrying a Korean citizen can help them to easily migrate to Korea. In many cases, foreign brides arrive in Korea shortly after marriage with little knowledge of their new spouses or their new country’s culture and language [5].

Actually, Korea has been considered as an ethnoracially homogeneous society for a long time. This myth was strengthened after the colonial period on the intention of making national cohesion and recovering national identity [6]. Thus, there is a widespread misconception that racism does not exist in Korea since it has long been known to be a mono-ethnic society. Due to U.S. military presence and its cultural influences after World War II, however, Korean society has accommodated the American idea of racism [7, 8]. The recent increased influx of immigrants in Korea has ignited racism among Korean natives, which is heightened by economic and cultural nationalism [6]. For example, more than 40 % of Koreans answered that they would not want a foreigner as their neighborhood, based on the recent World Value Survey (2010–2014) [9].

As Korea is becoming an ethnoracially diverse society, it is crucial to understand social implications of ethnic or racial discrimination against immigrants, particularly against marriage migrants for two reasons [10]. First, compared to other immigrant groups, marriage migrants are more likely to be legal (i.e., *de jure*) “Korean citizens” because they are naturalized and acculturated easily due to the marriage with native Koreans. However, social antipathy toward them suggests that they are *de facto* foreigners. Second, social hostility toward marriage migrants in Korea can lead to discrimination against their children as well as themselves, which is potential damage for social integration. Currently, more than 200,000 children of marriage migrants already reside in Korea [2], and they are often exposed to discrimination and bullying [11].

Ethnic or racial discrimination can negatively affect health. Indeed, the stress related to ethnic or racial discrimination can aggravate mental health such as depression [12–14], anxiety [12, 15], psychological distress [16], and somatization [15]. In addition, individuals who experience ethnic or racial discrimination can also exhibit physical symptoms including cardiovascular conditions [17], hypertension [18, 19], and high rates of pre-term birth [20]. Also in Asian countries, the adverse health effect of ethnic or racial discrimination has been examined recently. For example, a study for marriage-based immigrated women in Taiwan showed the association between racial discrimination and depressive symptoms [21].

Despite recent surveys indicating that Korea is among the most ethnoracially intolerant countries, ethnic or racial discrimination has not been well studied as a relevant social issue. For example, using a nationally representative dataset, a study assessed the prevalence of perceived discrimination in eight social situations and perceived reasons for discrimination. In that study, six different possible reasons (sex, education, age, disability, birth region, and others) were evaluated, but ethnicity/race was not included as a potential reason for discrimination in the questionnaire [22]. Recently, researchers have started to pay attention to how social injustices are associated with health conditions among marriage migrants and their family members in Korea [11, 23].

One controversial issue in the research on discrimination and health is gender difference. It is necessary to carefully consider gender when investigating the factors which could have an effect on health because of different social exposures by gender, as well as biological differences [24]. Previous studies have documented that gender could play an important role in the association between discrimination and health, suggesting that men and women should be considered separately when examining the association between discrimination and health [13, 25, 26]. For example, a study of 2,095 Asian immigrants in U.S. showed that women might have the lower threshold of discrimination which could aggravate their health [25].

Another relevant issue is the role of response against discrimination in the association between health outcomes and unfair treatments. Previous researches often classified responses to unfair treatment into active and passive coping strategies. Active coping refers to responses such as trying to protest or talking to others, whereas passive coping includes ignoring or acceptance [14, 20]. According to studies, active coping could buffer the adverse effect of unfair treatment on health compared to passive one [14, 20, 27, 28].

This study aimed to assess the prevalence of perceived ethnic discrimination and to examine the association between ethnic discrimination and self-rated health among marriage migrants in Korea, the main purpose of our study can be summarized by the following questions:

1. What is the association between perceived ethnic discrimination and self-rated health among marriage migrants in Korea? Does the association differ by the gender of marriage migrants?
2. Among the marriage migrants who experienced ethnic discrimination, what is the association between response to discrimination (i.e., whether or not asking for fair treatment) and self-rated health? Does the association differ by gender?

## Methods

### Data and study population

This study analyzed the data from ‘National Survey of Multicultural Families 2012’ (hereafter, NSMF), conducted by the Ministry of Gender Equality & Family and Statistics Korea. The NSMF is a nationally representative survey about multicultural families; in this research, multicultural families are confined as families with marriage migrants, their Korean spouses, and other family members. The aim of the NSMF is to understand the condition and position of multicultural families for the design and implementation of related policies. The NSMF can be obtained through the Korea Women’s Development Institute (http://*eng.kwdi.re.kr/*).

The target population of the NSMF was the family member of marriage migrants (i.e. marriage migrants, their spouses, and offspring), and non-marriage migrants naturalized in Korea. Data were collected in July 2012 through a two-step sampling process. In the first step, Statistics Korea identified the families that would be eligible for the survey. 850 districts were selected from the 3,470 total administrative districts that had at least one multicultural family. 26,098 families were then identified in the 850 administrative districts. Among those 26,098 families, 19,646 families were contacted. In the second step, data were collected through in-person interviews by trained personnel. And 15,341 families completed the survey interview (response rate 78.1 %). In sum, the responding families were made up of 15,001 migrants (12,531 female), 13,859 spouses, and 4,775 children. To focus on marriage migrants, we limited the study population to 14,406 (2,215 male) marriage migrants after excluding the participants with missing data (*N* = 222) or the non-marriage migrants (*N* = 373). This research received IRB exemption from the Office of Human Research Administration at the Korea University.

### Measures

#### Perceived ethnic discrimination & response to discrimination

Perceived ethnic discrimination was measured by the survey question, ‘Have you ever experienced any discrimination or neglect because you are a foreigner?’ Survey participants could answer ‘Yes’ or ‘No’. In this paper, the experience assessed by this question was considered as ethnic discrimination. This is because ‘foreigner’ means an ethnic or a racial minority, as well as a foreign national, considering the context of Korean society where only one ethnic group has been believed to exist [29].

Response to discrimination was assessed by whether or not they asked for fair treatment against the discrimination. So, the migrants with experience of ethnic discrimination was divided into two groups: ‘Asking for fair treatment’ group represents participants who asked for fair treatment against the discrimination, and ‘Not asking for fair treatment’ group represents migrants who did not ask for fair treatment against the discrimination.

#### Self-rated health

Self-rated health was measured by a survey question, ‘How is your overall health?’. Respondents could answer in a 5-Likert scale (1 very good, 2 good, 3 fair, 4 poor, and 5 very poor). The response was classified into two groups: one group with ‘good’ health ratings (responses 1 to 3) and the other group with ‘poor’ ratings (responses 4 and 5). Although a single measure of self-rated health cannot be sufficient to assess specific features of the health, previous studies have reported that the measure is a strong predictor of mortality across countries after adjusting for key covariates including co-morbidity and depression [30, 31].

### Confounders

Variables related to socio-demographics, socio-economic characteristics, and Korean fluency were included in the data analysis as potential confounders. Socio-demographic variables consisted of age, gender, education, marital position, nationality, years of being in Korea, and residential area. Age was divided into five categories (20–29, 30–39, 40–49, 50–59, and 60 years old or more). Gender was categorized as male and female. Education was divided into four groups: primary school or less, junior high school graduate, high school graduate, and college or more. Marital status was coded as currently married and previously married (widowed, divorced, or separated). NSMF assessed participant’s nationality into 16 categories: China, Japan, Taiwan, Mongolia, Vietnam, the Philippines, Thailand, Cambodia, Uzbekistan, South Asia (including Nepal, Bangladeshi, India, Sri Lanka, the Maldives, and Bhutan), East South Asia (including Indonesia, Malaysia, Myanmar, Singapore, and Laos), Russia, North America (including United States of America, and Canada), Western Europe (including Germany, England, Switzerland, Belgium, and Netherlands), Oceania (Australia and New Zealand), and other region. Years of stay in Korea was categorized into four groups: 1 year or less, 2–5 year, 6–9 year, and 10 year or more. Residential area was dichotomized into metropolitan area and rural area.

Socio-economic variables include income, occupation, and the family’s perceived socio-economic position in Korea. Monthly household income was categorized into four groups: very low (monthly average household income less than 1 million Korean won), low (monthly average household income between 1–2 million Korean won), middle (monthly average household income between 2–4 million Korean won), and high (monthly average household income more than 4 million Korean won). Occupation was classified into five categories based on employment status: permanent worker, precarious worker, employer, unpaid family worker, and unemployed [32]. Information on a family’s perceived socio-economic position was provided by the respondents, who could answer in an 11-Likert scale from the poorest families (1) to the wealthiest families (11). Responses were categorized into three groups: low (1–3), middle (4–8), and high (9–11) perceived socio-economic position.

Finally, Korean language ability was added as a potential confounder because the variable was expected to be associated with the experience of ethnic discrimination as well as the migrants’ health [33]. Korean language ability was assessed in four domains (i.e., speaking, listening, reading, and writing). For each domain, respondents could answer on a 5-Likert scale from very good (1) to very poor (5). Scores from each domain were added, giving a possible range from 4 to 20 points. The total summed score was classified into three categories: fluent (4–9), fair (10–15), and poor (16–20).

### Data analysis

Logistic regression was applied in two step analyses. First, we tried to examine the association between perceived ethnic discrimination and poor self-rated health after adjusting for potential confounders. Second, we examined the association between response to discrimination and poor self-rated health among the marriage migrants with the experience of ethnic discrimination (*N* = 5,880). In addition, we tried to check whether the associations differ by gender.

All statistical analyses were performed using STATA/SE version 13.0 (StataCorp, College Station, TX). Data are reported as percentages or odds ratios (ORs) with 95 % confidence intervals (CIs).

## Results

Table 1 presents the distribution of study population, prevalence of poor self-rated health, and perceived ethnic discrimination by socio-demographic, socio-economic, and Korean fluency variables. Overall prevalence of poor self-rated health was 8.8 % among marriage migrants. And migrant groups with higher age, lower education level, or low income showed higher prevalence of poor self-rated health. Migrants who identified their family as a low socio-economic position in Korea had a higher prevalence of discriminatory experience (50.1 %) than the other categories of perceived socio-economic position (39.3 % for the middle group; 33.7 % for the high group). Overall prevalence of perceived ethnic discrimination among the population was about 41 %.

**Table 1:** Distribution of study population, prevalence of poor self-rated health, ethnic discrimination, and asking for fair treatment among marriage migrants in South Korea (*N* = 14,406)

	Distribution	Poor self-rated health		Ethnic discrimination		Asking for fair treatment among the population who experienced ethnic discrimination^a^	
	N (%)	N (%)	*p*-value^b^	N (%)	*p*-value^c^	N (%)	*p*-value^d^
Gender			0.570		<0.001		<0.001
Male	2,215 (15.4)	201 (9.1)		1,021 (46.1)		431 (42.2)	
Female	12,191 (84.6)	1,061 (8.7)		4,859 (39.9)		1,467 (30.2)	
Age			<0.001		<0.001		<0.001
20-29	4,067 (28.2)	125 (3.1)		1,541 (37.9)		394 (25.6)	
30-39	4,855 (33.7)	260 (5.4)		2,101 (43.3)		717 (34.1)	
40-49	3,523 (24.5)	384 (10.9)		1,571 (44.6)		539 (34.3)	
50-59	1,330 (9.2)	251 (18.9)		513 (38.6)		189 (36.8)	
60-	631 (4.4)	242 (38.4)		154 (24.4)		59 (38.3)	
Education			<0.001		<0.001		<0.001
Primary school or less	1,202 (8.3)	200 (16.6)		428 (35.6)		114 (26.6)	
Junior high school graduate	2,688 (18.7)	288 (10.7)		1,038 (38.6)		294 (28.3)	
High school graduate	5,978 (41.5)	521 (8.7)		2,483 (41.5)		790 (31.8)	
College or more	4,538 (31.5)	253 (5.6)		1,931 (42.6)		700 (36.3)	
Marital status			<0.001		0.256		0.038
Currently married	13,735 (95.3)	1,054 (7.7)		5,592 (40.7)		1,789 (32.0)	
Previously married	671 (4.7)	208 (31.0)		288 (42.9)		109 (37.9)	
Years of stay in Korea			<0.001		<0.001		<0.001
≤ 1	176 (1.2)	8 (4.6)		30 (17.1)		4 (13.3)	
≤ 5	4,151 (28.8)	195 (4.7)		1,469 (35.4)		360 (24.5)	
≤ 9	4,190 (29.1)	351 (8.4)		1,851 (44.2)		616 (33.3)	
≥ 10	5,889 (40.9)	708 (12.0)		2,530 (43.0)		918 (36.3)	
Residential area			<0.001		<0.001		0.240
Metropolitan area	5,260 (33.9)	555 (10.6)		2,257 (42.9)		749 (33.2)	
Rural area	9,146 (66.1)	707 (7.7)		3,623 (39.6)		1,149 (31.7)	
Household income			<0.001		0.005		<0.001
Very low	1,427 (9.9)	370 (25.9)		530 (37.1)		152 (28.7)	
Low	4,265 (29.6)	436 (10.2)		1,770 (41.5)		543 (30.7)	
Middle	6,848 (47.5)	383 (5.6)		2,849 (41.6)		918 (32.2)	
High	1,866 (13.0)	73 (3.9)		731 (39.2)		285 (39.0)	
Perceived socioeconomic position			<0.001		<0.001		0.367
Low	3,144 (21.8)	518 (16.5)		1,575 (50.1)		496 (31.5)	
Middle	9,114 (63.3)	604 (6.6)		3,582 (39.3)		1,153 (32.2)	
High	2,148 (14.9)	140 (6.5)	<0.001	723 (33.7)		249 (34.4)	
Occupation					<0.001		<0.001
Permanent worker	2,739 (19.0)	137 (5.0)		1,229 (44.9)		496 (41.4)	
Precarious worker	3,724 (25.9)	317 (8.5)		1,821 (48.9)		581 (31.9)	
Employer	791 (5.5)	49 (6.2)		314 (39.7)		137 (43.6)	
Unpaid family worker	746 (5.2)	48 (6.4)		253 (33.9)		71 (28.1)	
Unemployed	6,406 (44.3)	711 (11.1)		2,263 (35.3)		613 (27.1)	
Korean language ability			<0.001		<0.001		<0.001
Fluent	6,547 (45.4)	725 (11.1)		2,486 (38.0)		947 (38.1)	
Fair	5,956 (41.3)	410 (6.9)		2,627 (44.1)		774 (29.5)	
Poor	1,903 (13.2)	127 (6.7)		767 (40.3)		177 (23.1)	

^a^Marriage migrants who asked for fair treatment among the migrants who had experienced ethnic discrimination

^b^ *p*-value of the Chi-square test comparing the prevalence of poor self-rated health across key covariates

^c^ *p*-value of the Chi-square test comparing the prevalence of ethnic discrimination across key covariates

^d^ *p*-value of the Chi-square test comparing the proportion of migrants who asked for fair treatment across key covariates among the population who experienced ethnic discrimination

Table 1 also shows the prevalence of asking for fair treatment against the discrimination among the discriminated migrants. Male marriage migrants (42.2 %) more asked for fair treatment compared to the female migrants (30.2 %) when they experienced ethnic discrimination. When migrant’s years of stay in Korea or age increased, the prevalence of asking for fair treatment also increased. Migrants with higher household income were more likely to ask for fair treatment. Permanent workers or employers more asked for fair treatment, whereas the unemployed or unpaid family workers showed low rate of asking for fair treatment against the discrimination. Also, prevalence of asking for fair treatment differed by Korean language ability. The marriage migrants with fluent Korean ability more asked for fair treatment when they experienced ethnic discrimination.

The overall association between ethnic discrimination and self-rated health is shown in Table 2. Ethnic discrimination was associated with poor self-rated health (OR: 1.49, 95 % CI: 1.31, 1.70) after adjusting for potential confounders. When we examined the association between ethnic discrimination and self-rated health separately for men and women, a gender difference in the association was observed. Perceived ethnic discrimination was associated with poor self-rated health only among female marriage migrants (OR: 1.53, 95 % CI: 1.32, 1.76).

**Table 2:** Association between ethnic discrimination and poor self-rated health among marriage migrants in South Korea (*N* = 14,406)

Experience of ethnic discrimination	Total					Male					Female				
		Unadjusted		Fully adjusted^b^			Unadjusted		Fully adjusted^b^			Unadjusted		Fully adjusted^b^	
	N	OR	95 % CI	OR	95 % CI	N	OR	95 % CI	OR	95 % CI	N	OR	95 % CI	OR	95 % CI
No	8,526	1	referent	1	referent	1,194	1	referent	1	referent	7,332	1	referent	1	referent
Yes^a^	5,880	1.34*	(1.12, 1.50)	1.49*	(1.31, 1.70)	1,021	0.84	(0.63, 1.13)	1.16	(0.81, 1.66)	4,859	1.46*	(1.59, 1.65)	1.53*	(1.32, 1.76)

*p < 0.001

*OR* odds ratio, *95 % CI* 95 % confidence interval

^a^Marriage migrants who experienced ethnic discrimination

^b^Adjusted for age, education, marital status, nationality, residential area, years of stay in Korea, income, occupation, the family’s perceived socio-economic position, and Korean language fluency

Furthermore, we examined the association between response to discrimination and self-rated health among marriage migrants who experienced discrimination after being stratified by their gender (Table 3). Among male marriage migrants, asking for fair treatment had negative association with poor self-rated health (OR: 0.61, 95 % CI: 0.36, 1.04) at the margin of statistical significance (*p* = 0.071). On the other hand, though it was also marginally short of significant after adjusting for potential confounders (*p* = 0.075), there was a positive association between asking for fair treatment and poor self-rated health among female marriage migrants (OR: 1.21, 95 % CI: 0.98, 1.50).

**Table 3:** Association between response to discrimination and poor self-rated health among marriage migrants who experienced ethnic discrimination in South Korea (*N* = 5,880)

Response to ethnic discrimination	Total					Male					Female				
		Unadjusted		Fully adjusted^c^			Unadjusted		Fully adjusted^c^			Unadjusted		Fully adjusted^c^	
	N	OR	95 % CI	OR	95 % CI	N	OR	95 % CI	OR	95 % CI	N	OR	95 % CI	OR	95 % CI
Not asking for fair treatment^a^	3,982	1	Referent	1	referent	590	1	referent	1	referent	3,392	1	referent	1	referent
Asking for fair treatment^b^	1,898	1.12	(0.94, 1.34)	1.1	(0.90, 1.34)	431	0.65	(0.41, 1.04)	0.61	(0.36, 1.04)	1,467	1.26*	(1.04, 1.53)	1.21	(0.98, 1.50)

*p < 0.05

*OR* odds ratio, *95 % CI* 95 % confidence interval

^a^Marriage migrants who experienced ethnic discrimination but did not ask for fair treatment

^b^Marriage migrants who experienced ethnic discrimination and asked for fair treatment against the discrimination

^c^Adjusted for age, education, marital status, nationality, residential area, years of stay in Korea, income, occupation, the family’s perceived socio-economic position, and Korean language fluency

## Discussion

Our study found that gender could play an important role in the association of health with ethnic discrimination and response to discrimination (Additional file 1: Table S1). There was a statistically significant association between ethnic discrimination and self-rated health only among female migrants. This finding goes along with the previous studies which show that poor health condition is strongly associated with discrimination among women compared to men [25, 34]. It might be hard for female marriage migrants to cope with unfair treatment properly because of their lack of social resources due to low socioeconomic status [14]. Therefore, ethnic discrimination could endanger the health of female marriage migrants in Korea. Among the study population, around 30 % of female marriage migrants completed college or more, whereas more than half of male migrants did. Similarly, 50 % of female marriage migrants were unemployed and only around 15 % of them were permanent workers, while 14.3 % were unemployed and 40.5 % were permanent workers among the males.

Another important finding from this study is the different association between asking for fair treatment and health in female and male marriage migrants. Contrary to male migrants who might benefit from asking for fair treatment, female marriage migrants who asked for fair treatment against the discrimination reported poorer self-rated health than those who did not ask for fair treatment. This finding is inconsistent with several previous studies reporting that active coping stratagies could attenuate adverse health effects of ethnic discrimination [20, 35, 36].

We interpreted the gender difference in the association with asking for fair treatment and health in two ways. First, when female migrants sought to ask for fair treatment against ethnic discrimination, it could be easily ignored by perpetrators, mainly Korean natives, compared to the asking of male migrants. It is possible that the perpetrators could consider female migrants as inferior to male migrants in Korean society, which reflects the influences of patriarchal and misogynistic ideology [37–40]. Therefore, their asking for fair treatment against discrimination, as an active response, might not be accepted by the perpetrators. This could aggravate health conditions of the female migrants, whereas the same behavior would not harm health of male migrants.

In addition, female migrants might resist only when facing unbearable situations, which could explain the strong association between asking for fair treatment and poor self-rated health among the female migrants who experienced ethnic discrimination. Previous studies reported that social minorities including women are more likely to internalize their inferior status and to accept unfair treatments [41, 42]. When we compared the prevalence of asking for fair treatment by gender, it was higher in the male migrants (42.2 %) than in the female migrants (30.2 %). These results correspond with previous studies which reported that women are less likely to behave with active copings compared to men [18, 43, 44].

This study has several limitations. First, we cannot rule out the potential reverse causation between ethnic discrimination and self-rated health due to the cross-sectional study design. However, a prospective study between discrimination and self-rated health affirmed the causal relationship between the two [45]. Second, there could be potential unadjusted confounders, such as previous health status, which could be associated with experience of ethnic discrimination and health conditions [26].

## Conclusions

To our knowledge, this is the first study to explore the association between response to ethnic discrimination and health in Korea. Despite the rapid increase in migrants in Korea, the toll that experience of ethnic discrimination takes on their well-being might have been poorly understood without the questions posed by this study. This study underlines the gender difference in the association between response to discrimination and poor self-rated health among the marriage migrants in Korea. Further studies are necessary to explain the gender difference in the association among the migrants in Korea considering the intersectionality of gender and ethnicity.

The findings suggest the urgent need to establish an anti-discrimination law for disadvantaged groups including immigrants in Korea. In 2015, UN Human Rights Committee already called for the registration of anti-discrimination law for migrants to the Korean government but it is still pending due to the lack of public consensus. Any policy only for immigrants, however, is not enough to protect their social rights. Not only ethnic discrimination against migrants is prevalent and seriously affects their health; but also ethnic discrimination can be intertwined with other inequalities including gender discrimination, as shown in our gender analysis. Therefore, an anti-discrimination law not just for migrants, but for all disadvantaged people, is needed to be established in Korea because of the intersectional nature of social inequalities based on race, ethnicity, gender, and class. Furthermore, policies for the empowerment of ethnic minorities are needed, and this could not only prevent social injustice including discrimination but also help them dealing with unfair situations.

## Abbreviations

NSMF, National Survey of Multicultural Families 2012

## Additional file

Additional file 1: Table S1. Association between perceived ethnic discrimination and poor self-rated health, and the role of response to discrimination among marriage migrants in South Korea (N=14,406). (PDF 99 kb)
