# Supplementary material for: Don’t ask for fair treatment? A gender analysis of ethnic discrimination, response to discrimination, and self-rated health among marriage migrants in South Korea
Source: Int J Equity Health. 2016 Jul 19;15:112. doi: 10.1186/s12939-016-0396-7 (PMC4949882; doi:10.1186/s12939-016-0396-7)
Supplement: Additional file 1: Table S1. — Association between perceived ethnic discrimination and poor self-rated health, and the role of response to discrimination among marriage migrants in South Korea (N=14,406). (PDF 99 kb) [file 12939_2016_396_MOESM1_ESM.pdf]

Table S1. Association between perceived ethnic discrimination and poor self-rated health, and the role of response to discrimination among marriage migrants in South Korea (N=14,406)

Experience and response to ethnic discrimination	Total					Male					Female				
		Unadjusted		Fully adjusted[d]			Unadjusted		Fully adjusted[d]			Unadjusted		Fully adjusted[d]	
	N	OR	95% CI	OR	95% CI	N	OR	95% CI	OR	95% CI	N	OR	95% CI	OR	95% CI
No experience[a]	8,526	1	referent	1	referent	1,194	1	referent	1	referent	7,332	1	referent	1	referent
Not asking for fair treatment[b]	3,982	1.29**	(1.13, 1.47)	1.43**	(1.24, 1.66)	590	0.99	(0.71, 1.39)	1.36	(0.91, 2.04)	3,392	1.35**	(1.17, 1.56)	1.42**	(1.21, 1.67)
Asking for fair treatment[c]	1,898	1.44**	(1.22, 1.70)	1.61**	(1.34, 1.94)	431	0.65*	(0.42, 0.99)	0.88	(0.54, 1.46)	1,467	1.71**	(1.43, 2.04)	1.77**	(1.45, 2.17)

*$p<0.05$, **$p<0.001$

OR, odds ratio; 95% CI, 95% confidence interval

[a] Marriage migrants who did not experience ethnic discrimination.

[b] Marriage migrants who experienced ethnic discrimination but did not ask for fair treatment.

[c] Marriage migrants who experienced ethnic discrimination and asked for fair treatment against the discrimination.

[d] Adjusted for age, education, marital status, nationality, residential area, years of stay in Korea, income, occupation, the family’s perceived socio-economic position, and Korean language fluency.
